# Management of a Lassa fever outbreak, Rhineland-Palatinate, Germany, 2016

**DOI:** 10.2807/1560-7917.ES.2017.22.39.16-00728

**Published:** 2017-09-28

**Authors:** Lutz Ehlkes, Maja George, Gerhard Samosny, Florian Burckhardt, Manfred Vogt, Stefan Bent, Klaus Jahn, Philipp Zanger

**Affiliations:** 1 Federal State Agency for Consumer & Health Protection Rhineland-Palatinate, Koblenz, Germany; 2 Postgraduate Training for Applied Epidemiology (PAE), Robert Koch Institute (RKI), Berlin, Germany; 3 European Programme for Intervention Epidemiology Training (EPIET), European Centre for Disease Prevention and Control (ECDC), Stockholm, Sweden; 4 These authors contributed equally to this article and share first authorship; 5 Health Department Alzey-Worms, Alzey, Germany; 6 Federal State Ministry for Social Affairs, Employment, Health, and Demographics Rhineland-Palatinate, Mainz, Germany; 7 Institute of Public Health, University Hospitals, Heidelberg, Germany; 8 Department of Infectious Diseases, Medical Microbiology and Hygiene, University Hospitals, Heidelberg, Germany

**Keywords:** disease outbreaks, disease transmission, infectious, hemorrhagic fevers, viral, communicable diseases, emerging, disease transmission, epidemiology, contact tracing, Arenaviridae

## Abstract

Due to rapid diagnosis and isolation of imported cases, community outbreaks of viral haemorrhagic fevers (VHF) are considered unlikely in industrialised countries. In March 2016, the first documented locally acquired case of Lassa fever (LF) outside Africa occurred, demonstrating the disease’s potential as a cross-border health threat. We describe the management surrounding this case of LF in Rhineland-Palatinate – the German federal state where secondary transmission occurred. Twelve days after having been exposed to the corpse of a LF case imported from Togo, a symptomatic undertaker tested positive for Lassa virus RNA. Potential contacts were traced, categorised based on exposure risk, and monitored. Overall, we identified 21 contact persons with legal residency in Rhineland-Palatinate: seven related to the index case, 13 to the secondary case, and one related to both. The secondary case received treatment and recovered. Five contacts were quarantined and one was temporarily banned from work. No further transmission occurred. Based on the experience gained during the outbreak and a review of national and international guidelines, we conclude that exposure risk attributable to corpses may currently be underestimated, and we present suggestions that may help to improve the anti-epidemic response to imported VHF cases in industrialised countries.

## Introduction

On 25 February 2016, a healthcare professional with a rapidly deteriorating health condition, was evacuated from Togo to Cologne, North Rhine-Westphalia, Germany, where he died of multiple organ failure within hours of hospital admission [1,2]. After an autopsy [3], the corpse was released from the hospital and transferred to a mortuary in Rhineland-Palatinate, where he was meant to be embalmed before repatriation to Togo. Six days after releasing the corpse, tissue samples that had been sent to the World Health Organization (WHO) Collaborating Centre for Arbovirus and Haemorrhagic Fever Reference and Research at the Bernhard Nocht-Institute for Tropical Medicine in Hamburg for further diagnostics tested positive for Lassa virus (LASV) [1]. As a result, contact tracing was initiated in all federal states of Germany where individuals might have been in contact with the patient while he was still alive or with his corpse.

Lassa fever (LF) is a viral haemorrhagic fever (VHF) caused by Lassa virus (LASV) – an enveloped, single stranded RNA virus belonging to the family of Arenaviridae. The incubation period ranges from 3 to 21 days [4]. Disease onset is characterised by unspecific, influenza-like symptoms. Signs of increased vascular permeability, such as haemorrhages, oedema, and shock, indicate severe LF [5]. Symptomatic treatment of LF comprises fluid replacement and antipyretic drugs. Intensive care is required for the management of severe LF. The nucleosid-analogon ribavirin inhibits viral replication and its early administration was shown to improve survival of patients with LF [6]. Its prophylactic use may be considered after exposure with high or very high risk of infection [7]. Due to the high proportion of inapparent and mildly symptomatic infections, overall mortality of LASV infection is ca 1%, but increases to 18–31% for cases that require hospital care [8,9]. There is currently no vaccine to prevent LV infection.

LASV is endemic in West Africa [10]. It is mainly transmitted via excretions of rodents, either by direct contact with the mucosae or breached skin, ingestion of contaminated food, or inhalation of contaminated dust. Its natural hosts are multimammate rats (*Mastomys natalensis*), which do not develop symptoms, but can excrete the virus for an extended period of time. In endemic regions, reported LASV seroprevalences in rodents range from 5% to 45% [11,12]. Distribution of the natural host is a strong determinant of LASV endemicity [10]. To date, human cases of LF have been reported from Benin, Burkina Faso, Côte d’Ivoire, Ghana, Guinea, Liberia, Mali, Nigeria, and Sierra Leone [10], where the population prevalence of LASV-specific IgG antibodies ranges from 14% to 44% [13,14]. Community outbreaks of LF are mainly fuelled by zoonotic transmission. For nosocomial outbreaks however, person-to-person transmission of LASV via bodily fluids is particularly relevant [15,16], and super-spreading events have been described [16].

Importations of LF cases to industrialised countries are generally rare, but could become a more common cross-border health threat considering increased connectivity to endemic countries. Although imported LF cases through international travel and repatriation have been reported from – among others - Germany, the United Kingdom (UK), and the United States (US) [17-21], there has been no report of secondary cases following the import to non-endemic parts of the world.

The Ebola virus disease (EVD) epidemic struck West Africa in 2014, and imported cases were reported from various industrialised countries. This illustrated the need for VHF outbreak management capacity to an unprecedented extent. The subsequent activities to scale up preparedness led to the compilation of various EVD outbreak management guidelines in Europe [22-25].

In Germany, there are two documents addressing the outbreak management of VHF: recommendations covering the outbreak management of VHF in general (published in 2001) [26], and another document addressing EVD in particular (triggered by the outbreak in West Africa and issued in March 2016) [22]. Both provide guidance and best practices with regard to diagnostics, treatment in dedicated facilities, biosafety, waste disposal, disinfection, logistics, contact tracing, risk classification, control measures, and post-process evaluation.

Here we describe the events surrounding a locally acquired case of LF outside of Africa and report the challenges we faced with regard to contact tracing and enforcement of control measures in the federal state of Rhineland-Palatinate. We then review national and international VHF guidelines to identify complementary information of value for managing VHF outbreaks in the respective setting. We also present suggestions that may help improve the anti-epidemic management of imported cases of VHF in Germany and Europe.

## Methods

### Contact tracing, exposure risk classification and management

Upon information about the laboratory confirmation of LV in the index case, we contacted the embalming contractor, on whose premises the corpse of the index case (IC) was stored, and asked for a list of employees and everyone else who could have been in contact with the corpse. We then followed up all potentially exposed individuals. For rapid initial assessment and due to time constraints, we conducted ad hoc interviews by phone, using a short standardised questionnaire to estimate the individual extent of exposure. Individuals reporting or suspecting having had contact to the corpse, to a potential symptomatic case, or to their bodily fluids, were considered contact persons and classified into risk categories according to the German VHF recommendations ([26], Box). We used the day of first exposure plus the minimum incubation period of 3 days as the earliest time point to produce secondary cases, in case symptom onset of a LF case and thus the beginning of the period of infectiousness could not be clearly discerned.

Box **Exposure risk classification of contacts to VHF cases, used in the Lassa fever outbreak in Rhineland-Palatinate, Germany, 2016^a^**

**Very high risk (Category Ia)**

• Subcutaneous contact *or* contact of a mucous membrane to bodily fluids *or* tissue of a VHF patient

**High risk (Category Ib)**

• Contact (skin or aerosol) to infectious bodily fluids/tissue of a VHF patient

• Contact to infectious bodily fluids/tissue/carcass of a VHF-positive animal

**Medium risk (Category II)**

• Providing care to or handling diagnostic samples of a VHF patient

• Direct contact to potentially contaminated objects of a VHF patient

• Direct contact to the corpse of confirmed or suspected VHF case

• Contact to VHF-positive animal

• Close proximity to a symptomatic VHF case, e.g. during flight

**Low risk (Category III)**

• Any other contact to a confirmed VHF case (e.g. in same room with VHF case)

• Contact of medical staff to confirmed case using proper personal protective equipment (including respirators)

VHF: viral haemorrhagic fever.
^a^ Translated from Fock et al. [26].

We initiated passive symptom monitoring of all contacts in the risk categories I-III. We asked them to report their body temperature and the occurrence of any symptoms by email or telephone every 12 hours for the maximum incubation period of 21 days post-exposure to the LF case. Depending on the assigned risk category, enhanced control measures were enforced (Table 1).

**Table 1:** Control measures for contacts of viral haemorrhagic fever cases, as used during the Lassa fever outbreak in Rhineland-Palatinate, Germany, 2016^a^

Exposure risk /Control measure	Contact is asymptomatic				Contact is symptomatic			
	Very high Ia	High Ib	Medium II	Low III	Very high Ia	High Ib	Medium II	Low III
**Symptom monitoring**	+	+	+	+	+	+	+	+
**Home quarantine**	NA	*	-	-	NA	NA	*	*
**Quarantine/isolation in hospital**	+	*	-	-	+	+	*	*
**Post exposure prophylaxis**	+	*	-	-	+	+	*	*
**Blood sample at baseline^b^**	+	+	-	-	NA	NA	NA	NA
**PCR diagnostics for LASV**	+	-	-	-	+	+	+	+
**Temporary work ban, high risk professions^c^**	+	+	*	-	+	+	+	+
**Temporary work ban, any profession**	+	*	-	-	+	+	*	*

+: measure is recommended; -: measure is not recommended; *: measure to be considered on individual basis; LASV: Lassa virus; NA: not applicable.

^a^ Measures based on reference [26].

^b^ For diagnostic testing, in case symptoms develop or disease progresses.

^c^ Professions with close physical contact to people (hospitals, schools, etc.); subject to individual decision.

Measures included 21 days of active symptom monitoring, temporary work ban, home/hospital quarantine, and/or isolation, post exposure. Other measures included PCR-diagnostics and post-exposure prophylaxis with ribavirin. For contacts who developed suspicious symptoms, a more cautious set of measures was applied. In some instances, this required individual review and decision making by a panel of experts in infectious diseases and public health.

After the first assessment, we conducted in-depth interviews of ca 30 minutes each, using a more qualitative approach with open questions. We started with contacts for whom initial underestimation of exposure risk was suspected, based on inconsistencies or missing information when triangulating information gathered during the ad hoc assessment. The interviewees were talked through the exposure incident and asked to describe the exact type and proximity of contact. The purpose of these interviews was to fill information gaps, clear inconsistencies, and identify further contact persons, but also to discuss the possibility for unconscious exposure, such as touching potentially contaminated surfaces or material. Based on our in-depth interviews, we re-assessed initial risk classifications and respective control measures (Table 2).

**Table 2:** Results of exposure risk categorisation based on ad hoc compared with in-depth interviews with contacts from a Lassa fever outbreak, Rhineland-Palatinate, Germany, 2016

Contacts	Ad hoc interview		In-depth interview / triangulation	
	Type of contact	Risk category^a^	Issues raised during reassessment	Risk category^a^
Index case	Nurse evacuated from Togo, died in Cologne, transferred to mortuary in Rhineland-Palatinate	NA	NA	NA
Contact 1 / secondary case	Handled corpse of IC with double gloving, no facial mask, no apron	III	Autopsied corpse of IC that was losing massive amounts of fluid; potential contact to contaminated surfaces or objects after ungloving	II
Contact 2	In same room as IC but no direct contact; work-related contact to C1	III	Potential contact to contaminated surfaces (door handle, sink) in room where C1 handled IC	II
Contacts 3–8	Team transporting corpse of IC to crematory; full personal protective equipment (BSL-4 equivalent)	III	No safety breach reported (upon probing)	III
Contacts 9–12	Potential exposure to bodily fluids of C1	Ib	No additional information obtained	Ib
Contact 13	Travelling in a car with C1	III	No contact to corpse or potentially contaminated surfaces; no direct contact to C1	III
Contacts 14–18	Visitors to C1	III	No additional information obtained	III
Contacts 19–21	C19 physical contact to C1 without personal protective equipment	II	Interviews with C19 led to identification of C20 and C21; had contact to C1 at onset of symptoms	II

BSL: biosafety level; C: contact; IC: index case; NA: not applicable.

^a^ According to viral haemorrhagic fever recommendations [26].

### Symptom monitoring and control measures

We enforced control measures according to German VHF recommendations [26] (Table 1). However, these do not explicitly specify which symptoms other than fever justify a more cautious set of measures. Therefore, we added other symptoms of interest as sore throat, rhinitis, headache, cough, other respiratory symptoms, mucosal bleeding, myalgia, arthralgia, and gastro-intestinal symptoms. We defined fever as oral, rectal, or tympanal body temperature ≥ 38.0 °C. The assessment of contacts was complicated by the seasonally high prevalence of influenza-like illnesses in March that lowered the already limited specificity of symptoms used to indicate potential LF. Therefore, in low risk contacts (III), we defined either fever or a reported progression in the intensity of non-febrile influenza-like symptoms (i.e. sore throat, headache, cough and/or rhinitis) over 4 days as the trigger for additional measures. Contacts with high or medium exposure risk (I-II), by contrast, were subject to enhanced measures, regardless of symptom progression, as soon as any of the above mentioned symptoms occurred. If indicated, genome level-informed [27] reverse transcriptase quantitative PCR from blood was performed at the biosafety level (BSL) 4 laboratory at the Institute of Virology, University of Marburg, to confirm LASV infection.

### Review of existing VHF guidelines

We searched governmental, European Union (EU), and WHO online resources for publicly accessible English and German language VHF/EVD guidelines of relevance for contact tracing and management in Europe. We reviewed the respective outbreak management sections for particular strengths and common gaps, and highlighted key points with potential benefit for the outbreak management following imported VHF cases.

### Ethical considerations

The events around this first LF outbreak outside Africa received wide media coverage. All details of identifiable subjects described in this work have been previously released into the public domain [1,2,28,29].

## Results

### Contact tracing, exposure risk classification and management

The autopsy results were not available until 6 days after the arrival of the corpse at the mortuary. By the time the PCR-positive test result was communicated, the embalmment had not yet been performed. However, two employees of the mortuary had potentially been exposed to infectious material. One contact (C1), an undertaker using double gloving but no facial protection and apron when handling the corpse, was classified as a risk category III contact in the initial rapid assessment. However, during the in-depth interviews we learned that the autopsied corpse had started to decompose and had lost large amounts of fluid when being handled. Talking C1 through this setting again, direct contact to bodily fluids could be excluded. However, considerable uncertainty about contact to contaminated surfaces after ungloving remained. Therefore, we re-classified C1 into risk category II (Table 2). The other contact (C2) was present in the same room as C1, but had no contact to the corpse (risk category III). Later during the outbreak investigation, similar doubts about having touched potentially contaminated surfaces as in the case of C1 arose. Hence, we also re-classified C2 from risk category III into II.

Two days after discovering the index case was LASV-positive, his corpse was transferred for cremation by a team in full personal protective equipment (BSL 4 equivalent). We classified them as risk category III contacts (C3–8) according to the VHF recommendations [26].

At the time of exposure to the corpse of the index case, C1 was recovering from an upper respiratory tract infection [1]. His condition worsened 4 days after handling the corpse. On day 6 post exposure, the LASV-positive test result of the index case was communicated to the health authorities. Being in risk category II, this warranted a home quarantine (from day 7 post exposure, onwards) and PCR-testing for LASV, although C1 had no fever at the time. On day 8 post exposure, the negative PCR test result of C1 was communicated. As symptoms of C1 still persisted, a second blood sample was taken. This tested PCR-positive for LASV on day 12 post exposure [27]. On the same day, the health status of C1 deteriorated and he was transferred to the isolation ward of Frankfurt University Hospital for treatment with ribavirin [29]. The patient fully recovered after 21 days in isolation.

At the time C1 tested positive for LASV, his deteriorating health justified the classification of those with likely exposure to his bodily fluids (C9-C12) into risk category Ib. We identified seven additional contacts to the secondary case, of which six (C13-C18) were classified into risk category III, and one (C19) into risk category II. Futhermore, in-depth interviews allowed us to trace two further contacts of C1, unnoticed previously (C20 and C21, both risk category II).

In total, we identified 21 contact persons with legal residency in Rhineland-Palatinate, Germany (Table 2): seven with contact only to the IC, 13 with contact only to the secondary case, and one contact with exposure to both cases. Compliance of all contacts during follow-up was high and we had no loss to follow-up. No further transmission occurred and the outbreak was declared over on 6 April 2016.

### Symptom monitoring and control measures

In total, nine contacts developed non-febrile influenza-like symptoms, particularly cough, rhinitis, and/or sore throat. Six of them were high or medium risk contacts (category Ib/II), and underwent PCR testing for LASV with negative results. High risk contacts (category Ib) were quarantined, offered post-exposure prophylaxis with ribavirin, and discharged 21 days post exposure. One medium risk contact (category II) was quarantined at home and one was subject to a temporary work ban. The three low risk contacts (category III) were closely monitored, but did not show any progression to more severe symptoms. Hence, neither PCR-testing nor additional control measures were enforced. A timeline of the outbreak is provided in the Figure.

**Figure:**
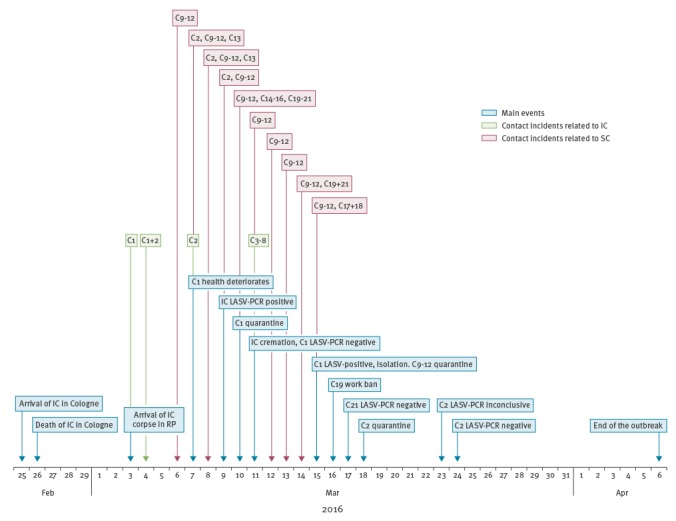
Timeline of events related to the Lassa fever outbreak, Rhineland-Palatinate, Germany, 2016

C: contact number; IC: index case; LASV: Lassa virus; SC: secondary case; RP: Rhineland-Palatinate.

Contact incidents before the minimum incubation period of 3 days are ignored.

### Review of existing VHF guidelines

In addition to the German VHF recommendations [26] used in the outbreak described here, we identified five guidelines of relevance: one concerning the management of VHF (UK) [30], and four concerning the management of EVD (Germany, European Centre for Disease Prevention and Control (ECDC), US Centers for Disease Control and Prevention (CDC) / WHO, and UK) [22-25]. Their approach is similar; first, an assessment of the exposure risk of suspected contacts is conducted. On the basis of the respective risk category and symptoms, control measures are enforced. In the context of the outbreak reported here, the review identified particular strengths but also gaps, which may be addressed in harmonised European guidelines for the management of VHF outbreaks in the future (Table 3).

**Table 3:** Strengths in VHF/EVD management guidelines relevant to Europe, 2016

Area of guidance	Strength	Example
Trigger for control measures	Comprehensive set of unambiguously defined symptoms, which allows taking reproducible decisions in favour of or against enforcing enhanced control measures	[22,24]
Exposure risk classification	Classify exposure to corpses of individuals who died of VHF, as high-risk	[22,23]
Personal protective equipment	Detailed description of the level of personal protective equipment required to decrease exposure risk	[22,23,30]
Interviewing contact persons	Guidance and suggestions on how to conduct interviews	[24]
Sexual transmission	Explicit mention of sexual transmission as an exposure risk (even after recovery) and how it can be avoided	[22-25,30]
Travel of contact persons	Explicit mention of travel restrictions as a control measure for contacts	[22,23,25,30]
Waste management	Instructions for waste disposal and disinfection measures	[22,30]
Information materials	Provision of template information sheet for contact persons	NA

EVD: Ebola virus disease; NA: not available; VHF: viral haemorrhagic fevers.

## Discussion

In 2014, the EVD epidemic quickly spread across borders and continents. As a result, most countries, including Germany, scaled up their EVD preparedness with a focus on timely detection of imported cases, as well as their isolation and treatment at designated facilities. This approach is considered most feasible and effective in preventing outbreaks, as detection of imported cases is expected before person-to-person transmission occurs.

Here we presented the first secondary case of LF outside Africa, occurring in an undertaker in Rhineland-Palatinate, after he came into contact with an autopsied corpse of a patient that had died from LF in Cologne, North Rhine-Westphalia, Germany. The abscence of similar cases and only one single published report of a physician seroconverting upon contact to a LF case [21] suggests that the overall risk of LF outbreaks triggered by imported cases is very low. Nevertheless, these observations illustrate that LASV transmission in the community and consecutive outbreak management in industrialised countries can become a realistic scenario. Being legally in charge of tracing contacts with residency in Rhineland-Palatinate, we faced a number of challenges that may also apply to similar settings in other countries, where VHF outbreaks in the community are currently an underestimated threat.

In outbreak situations, particularly those involving VHF, prioritisation of tasks and adequate time allocation are paramount. Ad hoc interviews allow an initial rapid assessment and provide an overview regarding the magnitude of the outbreak. However, our findings show that in-depth interviews with targeted probing for high risk situations and behaviours are essential during follow-up. These can lead to identification of further contacts and re-evaluation of initially assumed and potentially underestimated exposure risks. Interviews should also be employed to build trust between the investigators and the interviewees. Particularly in stressful situations, this will help enhance compliance and can thus be crucial for successful outbreak management. In this context, we considered the guidance provided by the CDC/WHO EVD management guideline [24] very helpful. For future guidelines, provision of template information material for contact persons, describing the disease as well as the rationale for control measures, would be welcome. Ideally, this material should be prepared together with experts in anthropology and psychologists with the aim to reduce fear and maximise compliance. From our experience, it should also contain information on the use of antipyretics. These were commonly used to treat the common cold by contact persons in the outbreak reported here, but can blur the onset of disease, and through prolonged inhibition of platelet aggregation may turn out to be detrimental in case of severe LF.

This investigation documents a considerable LASV exposure risk through contact to an autopsied corpse. In current guidelines reviewed in this investigation, the classification of exposure risk emerging from corpses is heterogeneous – as high risk [22,23], medium risk [26], while not being explicitly mentioned in others [25,30] – indicating the need for future harmonisation. Death from LF/VHF is the result of endothelial damage, capillary leak, shock, and multi-organ failure. Thus, corpses of patients that died of LF/VHF are likely to secrete fluids from edematous tissues, irrespective of the level of decomposition or whether they had undergone autopsy or not. This is particularly true in case of intensified volume therapy, fluid-overload, and advanced decomposition of the corpse – the latter of which was severe in our case. As the contact to corpses is thus associated with a high risk of being exposed to infectious bodily fluids, it would be helpful that harmonised guidelines classify contact to corpses of confirmed/suspected LF/VHF cases without full personal protective equipment, as high risk exposure at any rate.

During this outbreak investigation, the assessment of symptoms consistent with LF was complicated by the concurrent peak of the influenza season. To limit psychological stress for the affected contacts and their families, we had to sensibly adapt the threshold triggering enhanced control measures, particularly isolation and quarantine. Human case reports have measured detectable viraemia in blood on day 3 of symptom onset, whereas earliest detection of LASV in urine was not until 6–22 days after developing symptoms [31,32]. Viraemia and infectivity correlate with severity of symptoms [32]. Hence, a patient without severe symptoms is unlikely to be infectious through casual contact, particularly within the first 4 days of infection. The common cold typically lasts for a week, but shows no progression past day 3 of onset. We therefore decided to closely monitor all low risk contacts (III) that developed influenza-like symptoms, but no additional measures were enforced unless either a 4-day progression or more severe symptoms (including fever) occurred. For contacts with a high to medium exposure risk (I-II), any symptoms consistent with LF – even in absence of fever - triggered additional measures. Future guidelines may follow this example and employ a comprehensive operationalisation of how and when VHF-compatible symptoms/symptom progression trigger(s) control measures, with the goal to offer treatment as early as possible to those with a significant exposure risk, while reducing unnecessary stress for the involved parties. Ideally, future recommendations would also elaborate on the anatomical location(s) for temperature measurement that the fever threshold applies to.

In summary, our report provides lessons learned that can be employed to improve the response to imported VHF cases. Complemented by the strengths identified through a comparison of relevant guidelines, this experience may be reflected in harmonised European recommendations for VHF outbreak management. In particular, we suggest classifying the exposure risk attributable to corpses of individuals that had died of a VHF as high. As part of VHF preparedness in industrialised countries, more practical training of field epidemiologists in interviewing contacts and applying available tools for the classification of communal VHF exposure is needed.
